# Natural Occurrence of Deoxynivalenol and Its Acetylated Derivatives in Chinese Maize and Wheat Collected in 2017

**DOI:** 10.3390/toxins12030200

**Published:** 2020-03-22

**Authors:** Pianpian Yan, Zhezhe Liu, Shiqiao Liu, Liyun Yao, Yan Liu, Yongning Wu, Zhiyong Gong

**Affiliations:** 1 Key Laboratory for Deep Processing of Major Grain and Oil of Ministry of Education, College of Food Science and Engineering, Wuhan Polytechnic University, Wuhan 430023, China; pianpianyan1@gmail.com (P.Y.); liuzhezhewhpu@gmail.com (Z.L.); liushiqiaowhpu@gmail.com (S.L.); yaoliyunwhpu@gmail.com (L.Y.); liuyanwhpu@163.com (Y.L.); 2 Key Laboratory of Food Safety Risk Assessment, Ministry of Health, China National Centre for Food Safety Risk Assessment, Beijing 100021, China; wuyongning@cfsa.net.cn

**Keywords:** deoxynivalenol, 3-acetyl-deoxynivalenol, 15-acetyl-deoxynivalenol, co-occurrence mycotoxins, wheat and maize, UHPLC–MS/MS

## Abstract

Deoxynivalenol (DON), along with 3-acetyl-deoxynivalenol (3-ADON) and 15-acetyl-deoxynivalenol (15-ADON), occur in grains and cereal products and is often hazardous to humans and livestock. In this study, 579 wheat samples and 606 maize samples intended for consumption were collected from China in 2017 and analyzed to determine the co-occurrence of type-B trichothecenes (DON, 3-ADON, and 15-ADON). All the wheat samples tested positive for DON, while 99.83% of the maize samples were DON-positive with mean DON concentrations of 165.87 and 175.30 μg/kg, respectively. Per the Chinese standard limits for DON, 3.63% of wheat and 2.97% of the maize samples were above the maximum limit of 1000 μg/kg. The DON derivatives (3-ADON and 15-ADON) were less frequently found and were present at lower levels than DON in wheat. 3-ADON and 15-ADON had incidences of 13.53% and 76.40%, respectively, in maize. By analyzing the distribution ratio of DON and its derivatives in wheat and maize, DON (95.51%) was the predominant toxin detected in wheat samples, followed by 3.97% for the combination of DON + 3-ADON, while DON + 3-ADON + 15-ADON and DON + 15-ADON were only found in 0.17% and 0.35% of wheat samples, respectively. Additionally, a large amount of the maize samples were contaminated with DON + 15-ADON (64.19%) and DON (22.11%). The samples with a combination of DON + 3-ADON and DON + 3-ADON + 15-ADON accounted for 1.32% and 12.21%, respectively. Only one maize sample did not contain all three mycotoxins. Our study shows the necessity of raising awareness of the co-occurrence of mycotoxin contamination in grains from China to protect consumers from the risk of exposure to DON and its derivatives.

## 1. Introduction

Maize and wheat are widely cultured cereal crops and are considered staple foods in many regions around the world [1]. Global maize production in 2014 was 1037.8 million tons, mainly contributed by the United States, China, Brazil, the European Union, Argentina, Ukraine, India, and Mexico [2]. Wheat production in 2016 was 749 million tons worldwide, mainly harvested from China, India, Russia, and the United States [3]. In 2017, maize production in China was reported to reach 259 million tons, compared to wheat (134 million tons) and rice (214 million tons). China is a traditional agricultural country with maize and wheat always accounting for the important ratio of all food materials. However, approximately 25% of food worldwide is estimated to be contaminated by mycotoxins during growth, harvest, transportation, processing, or storage, resulting in yield losses and economic losses exceeding hundreds of billions of dollars [4]. 

*Fusarium* head blight (FHB) is a severe fungal disease that affects wheat and maize. The disease is caused by *Fusarium *graminearum** species complexes (FGSC) [5]. FHB-infected grains are often contaminated with a class of trichothecene mycotoxins [6]. These mycotoxins are produced by the genera *Fusarium graminearum* (*F. graminearum*)*, Fusarium asiaticum* (*F. asiaticum*)*, Fusarium meridionale,* and *Fusarium boothii* [7]. Trichothecenes are low-molecular-weight secondary metabolites and capable of causing disease and death in both humans and animals [8]. Researchers in many countries have surveyed the most important type B trichothecenes such as deoxynivalenol (DON) and its derivatives, especially for 3-acetyl-deoxynivalenol (3-ADON), 15-acetyl-deoxynivalenol (15-ADON), and deoxynivalenol-3-β-D-glucoside (D-3-G), which are considered to result in health issues [9]. Maize has been reported to be mainly contaminated by DON and zearalenone [10]. Similarly, DON and its derivatives have been suggested to have the greatest frequency in wheat [11].

The toxicological properties vary from compound to compound. The DONs (the sum of DON, 3-ADON, and 15-ADON) potentially cause chronic and acute toxicities, such as vomiting, hepatotoxicity, nephrotoxicity, neurotoxicity, and immunotoxicity [12,13]. In addition, severe vomiting resulting from DONs leads to alterations in the nutrient and electrolyte balance, thus threatening the health of humans and animals [14]. DON, 3-ADON and 15-ADON are capable of altering the intestinal barrier function of pigs by regulating the expression of tight junction proteins [15]. DON derivatives have been considered as markers for detecting DON in cereal crops because DON shows a co-occurrence with its derivatives [16]. According to an EU report in 2017, most 3-ADON and 15-ADON are de-acetylated into DON during absorption and distribution by animals [17]. Therefore, 15-ADON and 3-ADON have at least equal toxicity to animals compared to DON [18]. Humans are naturally and frequently exposed to multiple mycotoxins, which may act independently. However, most mycotoxins show synergistic effects with each other, which may have more severe impacts on human health [19].

Therefore, the acetylated forms of DON have attracted increasing attention. Researchers in other countries warned that contamination of DON and its derivatives, especially 3-ADON and 15-ADON, are significantly different compared with the maize grains found in different years [20]. The contamination level of DON derivatives in China is relatively high. With regard to animal studies and epidemiological studies of DON and its acetyl derivatives in humans, the Joint FAO/WHO Expert Committee on Food Additives (JECFA) established that the temporary maximum daily tolerable intake (PMTDI) for the sum of DON and its acetyl derivatives (3-ADON and 15-ADON) is 1 µg/kg BW/d. However, the National Criterion of China has established a maximum concentration (MLS) of DON in cereals and their products of 1000 µg/kg [21]. The maximum allowable legal limits of 3-ADON and 15-ADON have not yet been set [22,23]. The results obtained in this work are of great significance to food safety, especially since the literature on 3-ADON and 15-ADON contamination of food is relatively scarce in China. To our knowledge, no data have been published on the simultaneous presence of DON, 3-ADON, and 15-ADON in wheat and maize in 2017 from China. The contamination of mycotoxins in wheat and maize may pose a serious health threat to the local area. Consequently, the aim of this study is to evaluate the natural occurrence of the above mycotoxins in wheat and maize, as determined by UPLC–MS/MS described by GB 5009.111-2016 [24].

## 2. Results 

### 2.1. Optimization of the Clean-Up Procedure, Chromatographic Column, and Mass Spectrometry Conditions

**Figure 1:**
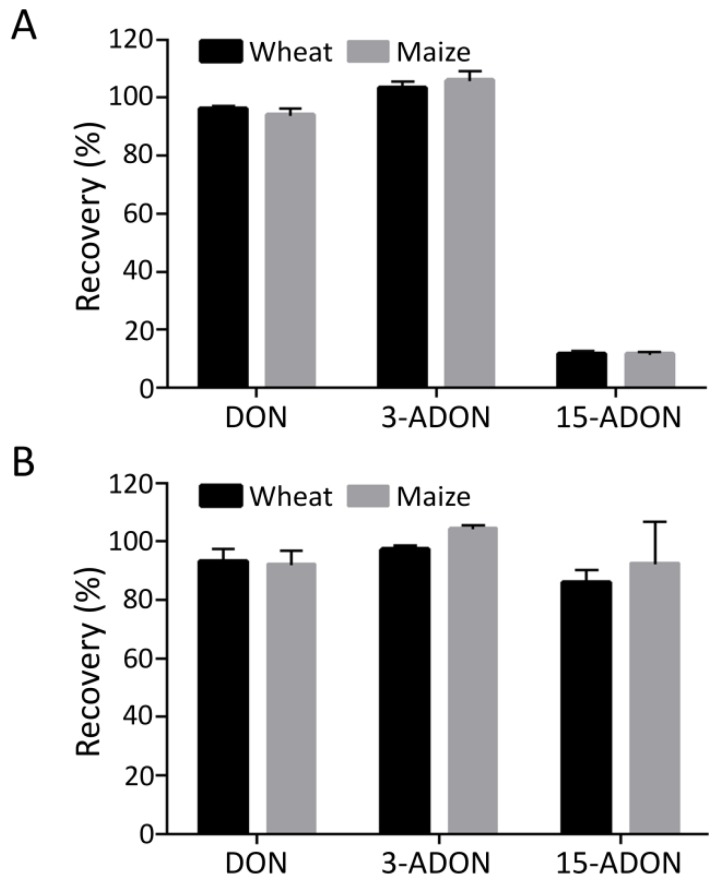
Recovery of maize and wheat spiked with deoxynivalenol (DON), 3-acetyl-deoxynivalenol (3-ADON), and 15-acetyl-deoxynivalenol (15-ADON) of immunoaffinity columns (IACs) (**A**) and multifunctional columns (MFCs) (**B**). The data are expressed as the mean + SD of three independent experiments.

Previous studies mainly described the use of immunoaffinity columns (IACs), multifunctional columns (MFCs), and solid phase extractions (SPEs) in the clean-up procedure [25,26,27]. Considering that a large number of samples and 3 types of mycotoxins needed to be analyzed in our research, we were unwilling to employ SPE because this method requires relatively complex procedures and takes a long time. Therefore, we evaluated IAC and MFC (Figure 1). The results showed that the MFC recovery rates of DON, 3-ADON, and 15-ADON in wheat were 92.90%, 97.12%, and 85.82%, respectively (Figure 1B). Additionally, the recovery rates of DON, 3-ADON, and 15-ADON in maize were 92.07%, 104.36%, and 92.44%, respectively (Figure 1B). However, the IAC recovery rates of 15-ADON in wheat and maize were 11.63% and 11.60%, respectively, suggesting that IAC is not suitable for application (Figure 1A).

To efficiently separate the three mycotoxins from samples of wheat and maize, we evaluated four high separation efficiency chromatographic columns (column a, Waters ACQUITY UPLC HSS (2.1 × 100 mm, 1.7 µm); column b, Waters ACQUITY UPLC BEH C18 (2.1 × 100 mm,1.8 µm); column c, Waters ACQUITY UPLC BEH C8 (2.1 × 100 mm, 1.8 µm); and column d, Waters ACQUITY UPLC BEH HILIC (2.1 × 100 mm, 1.8 µm; see Figure S1). The results show that the HSS column failed to separate DON from 3-ADON and 15-ADON effectively. With column b, clutter peaks appeared at 1 minute while column c had no retention for DONs. However, when applying column d, the peak shape was good, and almost no interfering peaks were observed, showing that DON, 3-ADON, and 15-ADON were effectively separated at 3.5 and 4.2 minutes, suggesting that column d is suitable for separating DON from 3-ADON and 15-ADON. Unfortunately, 3-ADON and 15-ADON were still unable to be effectively separated using ultra-high separation efficiency chromatographic columns because they are position isomers and differ from each other at the position of the acetyl groups on the carbon ring.

Therefore, to separate 3-ADON and 15-ADON from our grain samples, the specific selectivity of secondary mass spectrometry was applied to determine their masses based on their characteristic ion of different mass numbers in MRM mode (their mass numbers are 337.1 > 307 and 337.1 > 150, respectively). Specifically, the negative samples were labeled with 3-ADON and 15-ADON separately. When 3-ADON was added to the sample matrix, the 15-ADON ion channel did not show any interference. In contrast, the ion channels of 3-ADON had no interference with the addition of 15-ADON to the sample matrix, indicating that the specific selectivity of secondary mass spectrometry was suitable. The UPLC–MS/MS method was satisfactory and suitable for the simultaneous determination of DONs in wheat and maize (Figure S2).

### 2.2. Method Validation

Using our optimized procedure, we assessed the reproducibility and recovery according to the No. 401/2006 EU Regulation and the linearity, precision, detection limit, and quantitative limit based on the GB 5009.111-2016 [24,28]. The dilution ranges of DON, 3-ADON, and 15-ADON were from 5 to 500 µg/mL. The calibration curves were established by plotting the peak area (y) versus concentration (x) of each from UHPLC analysis (DON: y = 0.73x − 0.08; 15-ADON: y = 0.20x − 0.008; 3-ADON: y = 0.69x − 0.04). The obtained calibration curves of DON, 3-ADON, and 15-ADON were linear from 9.91–501.20, 6.97–500.50, and 4.93–500.05 µg/mL, respectively, which showed a correlation R^2^ of DON of 0.9996, while the correlation R^2^ values of both 3-ADON and 15-ADON were 0.9999. The estimation of the limits of detection (LODs) and limits of quantification (LOQs) was made by diluting the standard solution to their respective signal-to-noise ratio (S/N) of approximately 3 for the LOD and 10 for the LOQ. The LOD of DON, 3-ADON, and 15-ADON was 0.65, 0.20, and 0.20 µg/kg, respectively. In addition, LOQ was 2.18, 0.49, and 0.67 µg/kg, respectively. The recovery (R) and repeatability (relative standard deviation, RSD) of the method were checked for three fortification levels: 50, 100, and 200 µg/kg. According to the level of analyte, the obtained R-values ranged from 71.72% to 112.81% (Table 1), while the RSD values ranged from 2.26% to 13.98%. A DON-positive wheat and maize reference sample were extracted and analyzed 3 times within 1 day for inter-laboratory RSD calculation (Table S1). These results indicate that the analytical method displayed high accuracy and precision for the detection of DON, 3-ADON, and 15-ADON in wheat and maize samples.

### 2.3. Analysis of Samples

**Figure 2:**
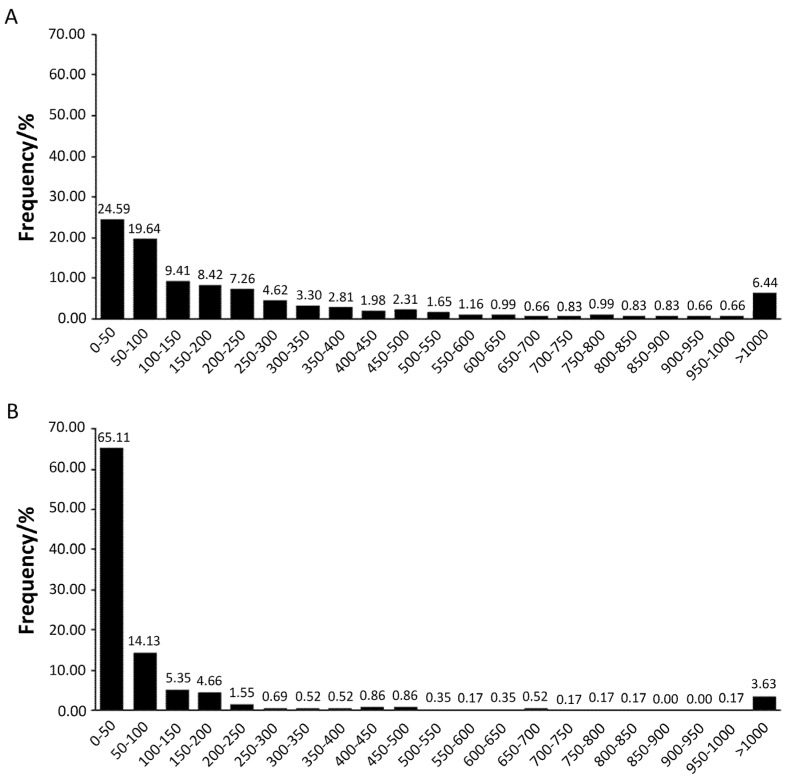
Distribution of DONs in wheat samples (**A**) and maize samples (**B**).

The results for the determination of DON and its derivatives in harvested wheat and maize kernel samples are presented in Table 2. DON showed frequencies of 100% and 99.83%, with mean concentrations of 165.87 and 175.30 μg/kg, ranging from 12.16 to 6436.11 μg/kg and not detected(ND) to 4300.7 μg/kg in wheat and maize, respectively. However, 3-ADON had a low incidence of 4.15% and 13.53%, with mean concentrations of 1.22 and 4.97 μg/kg, ranging from ND to 149.49 μg/kg and ND to 385.33 μg/kg in wheat and maize, respectively. The results of very low levels of 3-ADON in cereals, especially for wheat, are in line with previous studies [29]. Interestingly, 15-ADON presented a dramatically low incidence (0.52%) with a mean concentration of 0.20 μg/kg (ranging from ND to 24.46 μg/kg) in wheat, while it showed a high frequency of 76.40% with a mean concentration of 115.06 μg/kg (ranging from ND to 4811.06 μg/kg) in maize. Only one maize sample was free from the three mycotoxins. Based on the distribution pattern of DONs, most wheat and maize samples were contaminated by DONs, but 89.25% and 62.06% of maize and wheat samples were contaminated by DONs (<200 µg/kg), respectively (Figure 2). When compared to the maximum acceptable level (1000 μg/kg) in GB 2761, DON levels exceeding 1000 μg/kg in wheat and maize were 3.63% and 2.97%, respectively. However, 3-ADON was acceptable in both wheat and maize samples, while 15-ADON exceeded 1000 μg/kg at a rate of 0.50% of maize samples.

The regions with the main wheat-producing areas (Hebei, Henan, Jiangsu, Hubei, Anhui, and Xinjiang) and maize-producing areas (Jilin, Liaoning, Heilongjiang, Shandong, and Inner Mongolia) were selected for sampling. In the main wheat production areas, the frequency of DON contamination in Hebei, Henan, Jiangsu, Hubei, Anhui, and Xinjiang was 100% (Table 3). Among that, Xinjiang, Henan, Hubei, and Jiangsu provinces had 7, 2, 8, and 3 samples exceeding the national limit standard, respectively. The highest level of DON in Xinjiang exceeded the China national standard GB 2761 by more than 6.4-fold. The frequency of 3-ADON contamination in Hebei, Henan, Hubei, Anhui, and Jiangsu were 1.75%, 4.88%, 9.09%, 3.45%, 2.63%, respectively, which Xinjiang was in good condition. Liu et al. [30] reported that 3-ADON contamination in Hebei was 3.16% (mean concentration: 2.05 μg/kg), which was in accordance with this study. Samples from five provinces did not test positive for 15-ADON contamination, yet the frequency of 15-ADON contamination in Hebei was 27.27%.

In the main maize production areas, the frequency of DON contamination in Jilin, Liaoning, Inner Mongolia, and Shandong were all 100%, except for Heilongjiang, which showed 99.46% (Table 4). The frequency of 3-ADON contamination in Jilin, Liaoning, Heilongjiang, Inner Mongolia, and were 8.89%, 12.77%, 22.58%, 9.45%, respectively, while no sample was detected to exceed 1000 μg/kg. Additionally, a high fraction of the samples from all the provinces studied were 15-ADON-positive, with samples from Jilin, Liaoning, Heilongjiang, Inner Mongolia, and Shandong showing incidence rates of 72.22%, 71.28%, 77.96%, 80.31%, 100%, respectively. Li and Luo et al. [31,32] reported that maize was more severely contaminated with DON, 3-ADON, and 15-ADON compared to wheat.

### 2.4. Distribution of DON and Its Derivatives in Wheat and Maize from China

Concerning the contamination of DON, 3-ADON, and 15-ADON in the 2017 wheat samples examined, DON was the predominant toxin detected in wheat samples, followed by 15-ADON. DON showed a higher incidence of 95.51% and 3.97% with a combination of DON + 3-ADON, while DON + 3-ADON + 15-ADON and DON + 15-ADON were only found in 0.17% and 0.35% in the tested wheat samples, respectively. In addition, 4.49% of the samples were contaminated by at least two mycotoxins. However, we did not find any wheat samples containing 3-ADON and 15-ADON alone. Among 579 wheat samples, only one wheat sample contained the three toxins. It is noteworthy that the co-occurrence of the mycotoxins is quite common, especially in highly contaminated grains [33]. The largest 3-ADON (98.12 µg/kg) and 15-ADON (19.13 µg/kg) concentrations were quantified in the sample with a DON concentration of 5521.20 µg/kg. The concentration of DON exceeded the limit of the China national standard GB 2761 by more than 5-fold. Compared to the wheat samples, maize samples not only differed in the contamination levels but also in composition. A large amount of the maize samples were contaminated with DON + 15-ADON (64.19%) and DON (22.11%). The samples with a combination of DON + 3-ADON and DON + 3-ADON + 15-ADON accounted for 1.32% and 12.21%, respectively. Only one maize sample did not contain all the three mycotoxins. In addition, 77.72% of the maize samples contained two or more mycotoxins. Generally, whenever 3-ADON and/or 15-ADON were detected, DON was always present. However, we did not find any samples containing only a combination of 3-ADON and 15-ADON. Among the 39 (6.44%) maize samples that contained high levels of DON (exceeding the limits of the China national standard), 21 samples contained all the three mycotoxins. Considering that both 3-ADON and 15-ADON have similar toxicity as DON and are not included in the Chinese national standard regulations, it is extremely important to evaluate not only DON but all its derivatives [14,34]. 

## 3. Discussion 

DON, 3-ADON, and 15-ADON belong to the type-B trichothecenes produced by *Fusarium graminearum* and *Fusarium asiaticum*, which can cause hepatotoxicity, nephrotoxicity, neurotoxicity, and immunotoxicity in mammals [12,13]. DON, 3-ADON, and 15-ADON can contaminate agro-products such as wheat, maize, and even occur in agricultural commodities across the food chain. This is what has attracted public health attention.

The contamination trends of DON and its acetylated derivatives in wheat and maize analyzed in our study is consistent with a previous study which reported that DON was more frequently present in grains than 15-ADON and 3-ADON [23]. When compared with the data from 2011 maize kernels from 24 Chinese provinces, the prevalence of DON, 3-ADON, and 15-ADON in 2017 maize samples were significantly lower than that in 2011 maize samples (100%, 33%, 67%, respectively) [20]. Also, the average levels of DON, 3-ADON, and 15-ADON in 2017 maize samples were 175.3, 5.0, and 115.1 µg/kg, respectively, compared to 119, 1, 28 µg/kg, respectively, in the 2011 maize samples. Additionally, an earlier study in 2010 showed that the prevalence of DON, 3-ADON, and 15-ADON in wheat samples were 96.8%, 64.0%, 95.2%, respectively, implying that the incidence of 3-ADON and 15-ADON in the 2017 wheat samples had reduced significantly [35]. These differences in prevalence could be due to climatic conditions and geographical locations where the samples were collected [36]. The DON derivatives (3-ADON, 15-ADON) were far less frequently found and were present at low levels in wheat. However, 3-ADON and 15-ADON had incidences of 13.53% and 76.40% in maize, respectively. This is similar to the results reported that high incidence and concentrations of 15-ADON in maize samples than in wheat samples around the same period [37]. 

The contamination of DON, 3-ADON, and 15-ADON showed regional differences (Table 3 and Table 4). The mean concentrations of DON in maize samples collected in Jilin, Liaoning, and Heilongjiang provinces were above 180 µg/kg and had 5, 4, and 9 samples exceeding the national limit standard, respectively. There may be other factors such as insect infestation and agricultural practices affecting *Fusarium* infection. We found that wheat samples from five provinces did not contain 15-ADON except for Hubei Province (27.27%). Additionally, the mean content and standard-exceeded rate of DON was highest (72.73%) in wheat samples from Hubei. Our results agree with Zhang et al. [38], who reported that all wheat samples in Hubei contained high levels of DON, with 85.71% of the positive samples containing higher toxin levels than the Chinese national standard GB 2761 (1000 μg/kg). The incidence of 3-ADON was 21.43%. According to Waalwijk et al. [39], the DON and 3-ADON-producing fungus *F. asiaticum* was exclusively present in fields in the southern Hubei Provinces. The region has high temperature and humidity, and hence the fungal growth is favored during the period of flowering, sprouting, and heading stages. This may account for the high *Fusarium* infection and DON prevalence along the middle and lower regions of the Yangtze River [19,35,40,41]. 

**Figure 3:**
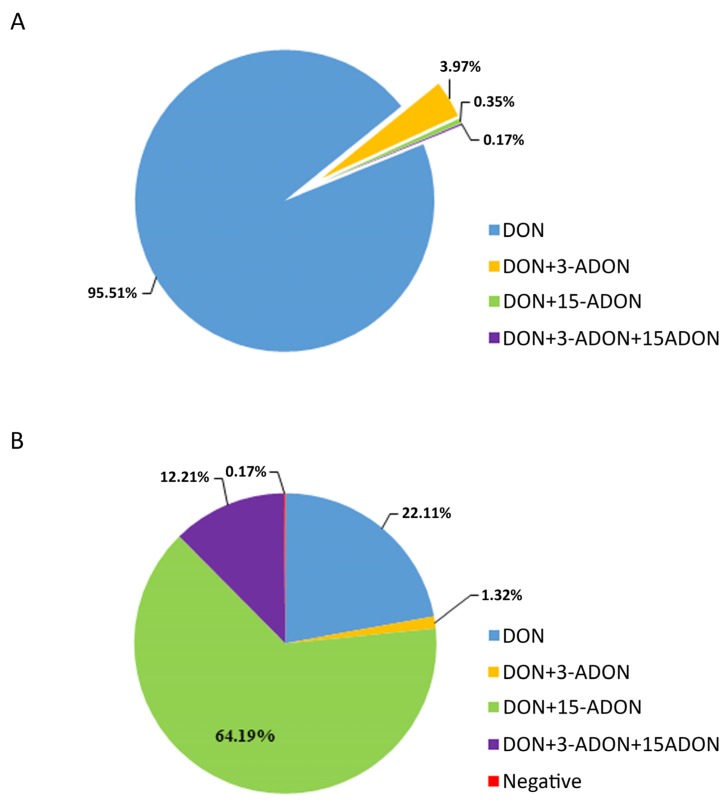
The distribution of wheat samples (**A**) and maize samples (**B**) according to the composition of mycotoxins.

According to Speijers [42], most mycotoxins do not only act alone but also act synergistically with other mycotoxins, which may be more harmful to human health. Mycotoxin compositions differed between the cereals (Figure 3). Our results in this study indicate a low incidence of 15-ADON (3.97%) and 3-ADON (0.35%) co-occurrence in the wheat samples. Similarly, Li et al. [31] did not detect DON + 3-ADON in their wheat samples. Janaviciene et al. [33] reported that the prevalence of deoxynivalenol and its derivatives in spring wheat grains from different agricultural production systems were different. A similar reason may account for why different levels of mycotoxins were detected in the cereals we collected from the different provinces in this study. It is known that the composition of mycotoxin chemotypes is related to pathogen species [43]. *F. graminearum* is mainly found in the cool northern areas of China with an annual average temperature below 15 °C, while *F. asiaticum* is present in warm southern areas with an annual average temperature above 15 °C [43,44,45]. Most *F. asiaticum* are 3-ADON producers, while *F. graminearum* produces 15-ADON chemotypes. In this study, all the maize samples were collected from the cool north of China, and that may account for why the incidence of DON + 15-ADON contamination was high (64.19%). 

Taken together, the study demonstrates a natural occurrence of DON and its acetylated derivatives and the co-occurrence of three type-B trichothecenes from various regions of China in 2017. The results of the present study suggest different levels of DON, 3-ADON, and 15-ADON contamination in maize and wheat. Diversities in DON, its derivatives, and their compositions were found in wheat and maize samples in which 5 combinations of B-type trichothecenes were detected. The co-occurrence of DON + 15-ADON in maize kernels in China should be considered significant because of their high incidence in the grains. Further, in view of the cumulative health risks of the DON derivatives, it is indeed essential to appeal for a thorough reform of legislation, focusing not only on the original forms of mycotoxins but also on their derivatives. 

## 4. Materials and Methods

### 4.1. Chemicals, Reagent, Standards, and Instruments

All organic solvents, including acetonitrile and methanol used for sample extraction and ultra-performance liquid chromatography–tandem mass spectrometry (UPLC–MS/MS) analysis, were LC/MS grade and purchased from Fisher Scientific. Stock standard solutions of DON, 3-ADON, and 15-ADON, in acetonitrile with concentrations of 100.0, 100.0, and 100.0 mg/L, respectively, were purchased from Romer-labs (Tulln, Austria) and stored at −20 °C. ^13^C-DON (stable isotope-labelled internal standard, 50.0 µg/ml) and ^13^C-3-ADON (stable isotope-labelled internal standard, 25.0 µg/ml) stock solutions in acetonitrile were purchased from Romer-labs (Tulln, Austria). The working standard solutions were prepared in the mobile phase and kept at 4 °C. Wheat and maize reference materials for DON were purchased from Biopure (Tulln, Austria). Purified water was obtained from a Milli-Q system (Millipore, Bedford, MA, USA). Separation of the analytes was achieved on a Waters ACQUITY UPLC BEH HILIC (2.1 × 100 mm, 1.8 µm) column. The identification of mycotoxins was performed on a 6460–1290 ultra high performance liquid chromatography mass spectrometer. The 0.22-µm pore organic phase filter membrane was purchased from Jinteng (Tianjin, China). A Mycosep 226 multi-functional purification column was purchased from Romer-labs (Tulln, Austria).

### 4.2. Sample Collection 

**Figure 4:**
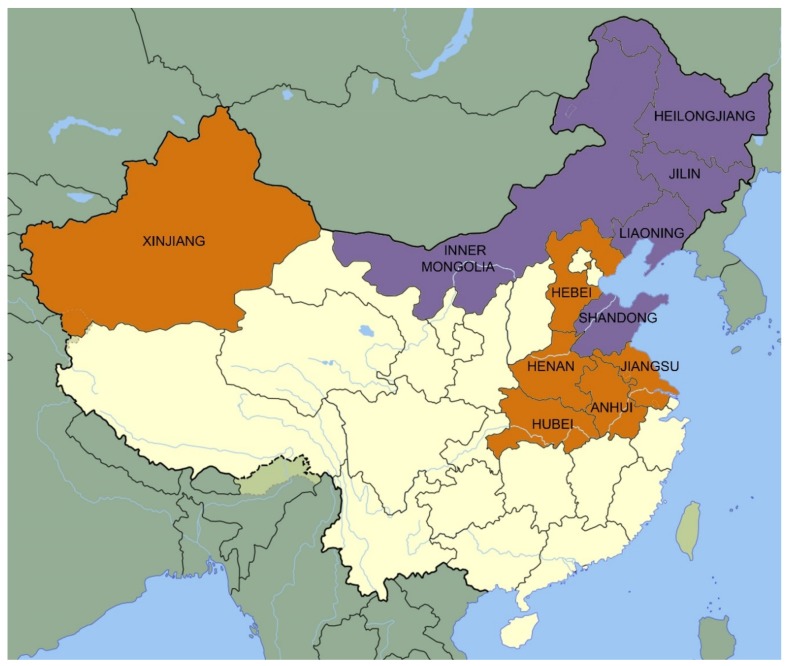
Distribution map of sampling areas (orange color: wheat samples; purple color: maize samples).

A total of 579 wheat kernel samples were collected from the main wheat-producing provinces (Hebei, Henan, Jiangsu, Hubei, Anhui, and Xinjiang) and a total of 606 kernel maize samples were collected from the main maize-producing provinces (Jilin, Liaoning, Heilongjiang, Shandong, and Inner Mongolia), shown in Figure 4. All wheat and maize kernel samples in 2017 intended for people consumption were collected from local farmers, one portion per household. The weight of each sample is 1 kg. The samples were stored at 4 °C until analysis.

### 4.3. UHPLC–MS/MS Conditions

UHPLC–MS/MS was implemented on an Agilent 1290-6460 system (CA, USA) equipped with a Z-spray ESI interface. UHPLC separation was conducted with a Waters Acquity UPLC BEH HILIC column (2.1 × 100 mm, 1.8 µm) using a mobile phase consisting of acetonitrile (A) and 0.2% ammonium hydroxide in water (B) at a flow rate of 0.3 mL/min. The gradient elution was applied as follows: 5%−20% B (0–1.5 min), 20%–40% B (1.5–3.0 min), 40%–90% B (3.0–5.0 min), 90%–20% B (5.0–7.0 min), 20%–5% B (7.0–8.0 min), and then returned to the initial composition within 1 min and equilibrated for 2 min before successive sample injection. The column was kept at 35 °C, and the injection volume was 5 µL. The ESI interface was operated in a negative-ionization mode. Nitrogen was used for desolvation, and its flow rate was 900 L/h. The desolvation temperature was 350 °C. The specific MS parameters of the compound, together with the product ions and precursors used for quantification and qualification, are shown in Table 5.

### 4.4. Sample Preparation and Clean-Up

Both wheat and maize samples were screened for dust and some minor impurities through a circular sieve with a diameter of 1.0 mm. The three mycotoxins DON, 3-ADON, and 15-ADON were extracted and cleaned up by the method described (GB 5009.111-2016). After milling, cereal samples (2 g) were extracted with 20 mL of acetonitrile/water (84:16, v/v) for 20 min on an orbital shaker and centrifuged at 10,000 rpm for 5 min. A 9-mL portion was passed through a MycoSep 226 column, and then 5 mL of purified extract was evaporated to dryness at 40 °C under a nitrogen flow. The residue was completely dissolved in 1 mL of methanol-water (95/5, v/v) with vortexing for 1 min. The mixture was homogeneous for 10 seconds with a scroll mixer and filtered into a 1.5-ml centrifugal tube with a 0.22 µm microporous filter membrane. A 180-µL sample and 20 µL of mixed isotope internal standard solution were absorbed into the injection bottle and analyzed by UHPLC–MS/MS for DON, 3-ADON, and 15-ADON. All maize and wheat samples were tested in Hubei Provincial Center for Disease and Prevention during the period February to July 2018.

### 4.5. Quantitation and Calculation

Quantitation of DON and its derivatives in the solution should be performed by measuring the peak area of the target compound and comparing it with the corresponding standard chromatographic peak in a change range of 2.5%. DON is analyzed with ^13^C-DON as an internal standard, and 3-ADON and 15-ADON are corrected with ^13^C-3-ADON as an internal standard. Analysis of naturally contaminated samples and wheat and maize reference materials were performed using ^13^C-labelled mycotoxins as internal standards added to the final samples according to the above-described procedure. The DON (3-ADON, 15-ADON) peak area was compared with the peak area of the relevant ^13^C-labelled internal standard. The formula for the mycotoxin content should be used to calculate the concentration of mycotoxins in the test sample:

$$x=\rho \times V_{1}\times V_{3}/V_{2}\times m$$

where ρ is the concentration of DON (3-ADON, 15-ADON) derived from the calibration curve (in micrograms/milliliter), *V_1_* is the extraction volume of maize and wheat sample (in milliliters), and *V_3_* is the final volume (in milliliters) of the injected test solution. *V_2_* is the volume (in milliliters) of the extract used for clean up, and *m* is the mass of the sample substance used for analysis in maize or wheat (in grams).

### 4.6. Statistical Analysis

All the data were analyzed by EXCEL 2007 and SPSS21.0. The average number of samples, mean ± relative standard deviation (RSD) + standard deviation (SD) and quartile (P_25_–P_75_) were calculated. When calculating the average value of data, the content value of an analysis result lower than LOD was replaced by LOD/2 [46]. 

## Figures and Tables

**Table 1:** Method validation parameters for the quantification of DONs in wheat and maize by the proposed UHPLC–MS/MS method.

Type	Compound	Natural Contamination	Spike Level	Accuracy Recovery	Precision (RSD)	
		(*n* = 6, µg/kg)	(µg/kg)	(%)	Interday (*n* = 6, %)	Intraday (*n* = 3, %)
	DON	14.13	50.00	100.96	10.37	9.23
			100.00	81.08	5.73	4.92
			200.00	82.34	6.61	10.44
wheat	3-ADON	ND^1^	50.00	104.44	3.76	3.98
			100.00	101.31	6.25	7.81
			200.00	82.50	2.95	7.97
	15-ADON	ND	50.00	103.15	4.10	10.47
			100.00	83.83	12.27	10.01
			200.00	71.72	5.36	6.07
	DON	16.45	50.00	93.50	10.52	7.94
			100.00	90.76	5.53	7.68
			200.00	92.25	5.71	7.89
maize	3-ADON	ND	50.00	103.93	7.80	6.87
			100.00	101.65	13.98	10.3
			200.00	91.72	2.83	4.12
	15-ADON	ND	50.00	112.81	2.26	8.62
			100.00	79.26	4.63	4.63
			200.00	74.55	8.62	6.43

^1^ ND-Not detected (<LOD).

**Table 2:** Total content of DON and its derivatives in wheat and maize in China in 2017 in this study.

Crops	Compound		Concentration (µg/kg)					≥1000 µg/kg/%
		*n*	Range	Mean	P_25_–P_75_	Median	Incidence/%	
Wheat	DON	579	12.16–6436.11	165.87	20.68–72.83	32.37	100	3.63
	3-ADON	579	ND^1^-149.49	1.22	ND	ND	4.15	-
	15-ADON	579	ND−24.46	0.20	ND	ND	0.52	-
	DONs	579	12.16–6436.11	167.30	21.05–75.20	32.74	100	3.63
Maize	DON	606	ND−4300.7	175.30	18.80–161.20	37.7	99.83	2.97
	3-ADON	606	ND−385.33	4.97	ND	ND	13.53	-
	15-ADON	606	ND−4811.06	115.06	15.80–152.20	58.30	76.40	0.50
	DONs	606	ND−5036.87	295.33	50.45–323.24	131.74	99.83	6.44

^1^ ND-Not detected(<LOD).

**Table 3:** Frequency and levels of DON and its derivatives of wheat collected in China in 2017 in this study.

Region	Compound		Concentration (µg/kg)					≥1000 µg/kg/%
		*n*	Range	Mean	P_25_–P_75_	Median	Incidence/%	
Hebei	DON	114	12.16–230.02	45.80	20.42–49.31	28.6	100	0
	3-ADON		ND^1^ −11.23	0.28	ND	ND	1.75	0
	15-ADON		ND	0.10	ND	ND	0	0
	DONs		12.16–230.02	46.18	20.42–49.31	28.60	100	0
Xinjiang	DON	59	14.54–6436.11	455.44	26.91–388.99	87.38	100	11.86
	3-ADON		ND	0.10	ND	ND	0	0
	15-ADON		ND	0.10	ND	ND	0	0
	DONs		14.54–6436.11	455.64	26.91–388.99	87.38	100	11.86
Henan	DON	328	12.37–1342.51	60.33	19.71–50.13	27.07	100	0.61
	3-ADON		ND −149.50	1.41	ND	ND	4.88	0
	15-ADON		ND	0.1	ND	ND	0	0
	DONs		12.37–1342.51	61.84	19.86–52.20	29.00	100	0.61
Hubei	DON	11	413.15–5521.20	2670.58	674.72–4637.08	2848.90	100	72.73
	3-ADON		ND−98.12	9.01	ND	ND	9.09	0
	15-ADON		ND−24.46	5.29	ND−13.77	ND	27.27	0
	DONs		413.15–5638.45	2684.87	674.72–4637.08	2848.90	100	72.73
Anhui	DON	29	15.36–2503.08	149.73	21.95–77.51	37.26	100	3.45
	3-ADON		ND−12.28	0.52	ND	ND	3.45	0
	15-ADON		ND	0.10	ND	ND	0	0
	DONs		15.36–2503.08	150.34	21.95–80.64	37.26	100	3.45
Jiangsu	DON	38	17.53–1817.96	270.79	42.96–287.17	99.41	100	7.89
	3-ADON		ND−42.83	2.41	ND	ND	2.63	0
	15-ADON		ND	0.10	ND	ND	0	0
	DONs		17.53–1817.96	273.30	42.96–287.17	102.21	100	7.89

^1^ ND-Not detected(<LOD).

**Table 4:** Frequency and levels of DON and its derivatives of maize collected in China in 2017 in this study.

Region	Compound		Concentration (µg/kg)					≥1000 µg/kg/%
		*n*	Range	Mean	P_25_–P_75_	Median	Incidence/%	
Jilin	DON	180	13.78–4300.69	224.17	27.00–232.56	75.28	100	2.78
	3-ADON		ND^1^−385.33	5.15	ND	ND	8.89	0
	15-ADON		ND−4811.06	182.20	ND−234.01	109.77	72.22	1.11
	DONs		16.53–5036.87	411.53	106.50–474.66	210.47	100	10
Liaoning	DON	94	14.47–4258.12	210.97	22.33–163.96	46.53	100	4.26
	3-ADON		ND−41.59	2.99	ND	ND	12.77	0
	15-ADON		ND−775.64	76.19	ND−97.75	37.36	71.28	0
	DONs		17.67–4258.12	290.15	40.29–262.30	82.47	100	6.38
Heilongjiang	DON	186	ND−2460.31	197.14	18.98–191.80	43.10	99.46	4.84
	3-ADON		ND−258.94	8.41	ND	ND	22.58	0
	15-ADON		ND −1032.94	86.45	16.63–102.13	43.49	77.96	0.54
	DONs		ND −3752.19	292.01	39.03–311.88	102.87	99.46	8.06
Inner Mongolia	DON	127	14.53–747.23	61.43	16.73–34.52	18.61	100	0
	3-ADON		ND−27.56	1.87	ND	ND	9.45	0
	15-ADON		ND−596.76	99.73	24.39–176.59	46.63	80.31	0
	DONs		14.59–984.49	163.03	49.67–241.71	84.14	100	0
Shandong	DON	19	16.30–659.38	83.22	21.61–77.05	77.05	100	0
	3-ADON		ND	0.10	ND	ND	0	0
	15-ADON		13.71–244.86	53.69	29.38–52.58	52.58	100	0
	DONs		30.01–904.23	137.01	59.35–138.59	138.59	100	0

^1^ ND-Not detected(<LOD).

**Table 5:** Optimised ESI–MS/MS parameters.

Compound Name	Precursor Ion (m/z)	Product Ion (m/z)	Fragmentor eV	Collision Energy eV	Retention Time (min)	LOD (µg/kg)	LOQ (µg/kg)
DON	295.1	265.1^1^	86	8	3.5	0.65	2.18
		138	86	16	3.5		
3-ADON	337.1	307^1^	86	8	4.2	0.20	0.49
		173	86	8	4.2		
15-ADON	337	150^1^	81	21	4.2	0.20	0.67
		219	81	9	4.2		
^13^C-DON	310.2	279.1^1^	91	8	3.5		
^13^C-3-ADON	354	323.1^1^	86	8	4.2		

^1^ Quantifier ion.

## Supplementary Materials

The following are available online at https://www.mdpi.com/2072-6651/12/3/200/s1, Figure S1: Chromatographic charts of three toxins separated by four chromatographic columns (column a, column b, column c, column d), Figure S2: 3-ADON (**A**) and 15-ADON (**B**) chromatograms for negative samples, Table S1: Concentration and precision of material references.
