# Supplementary material for: Natural Occurrence of Deoxynivalenol and Its Acetylated Derivatives in Chinese Maize and Wheat Collected in 2017
Source: Toxins (Basel). 2020 Mar 22;12(3):200. doi: 10.3390/toxins12030200 (PMC7150931; doi:10.3390/toxins12030200)
Supplement: Supplementary file 1 [file toxins-12-00200-s001.pdf]

Supplementary Materials: Natural Occurrence of Deoxynivalenol and Its Acetylated Derivatives in Chinese Maize and Wheat Collected in 2017

Pianpian Yan, Zhezhe Liu, Shiqiao Liu, Liyun Yao, Yan Liu, Yongning Wu, and Zhiyong Gong

Table S1. Concentration and precision of material references.

**Times**	**DON Concentration (μg/kg)**	
	**Maize**	**Wheat**
1	513.90	890.35
2	542.83	989.30
3	531.12	857.74
4	540.76	886.89
5	537.19	914.73
6	539.35	893.82
Average	534.19	905.47
Standard deviation	10.72	44.95
RSD	2.01%	4.96%

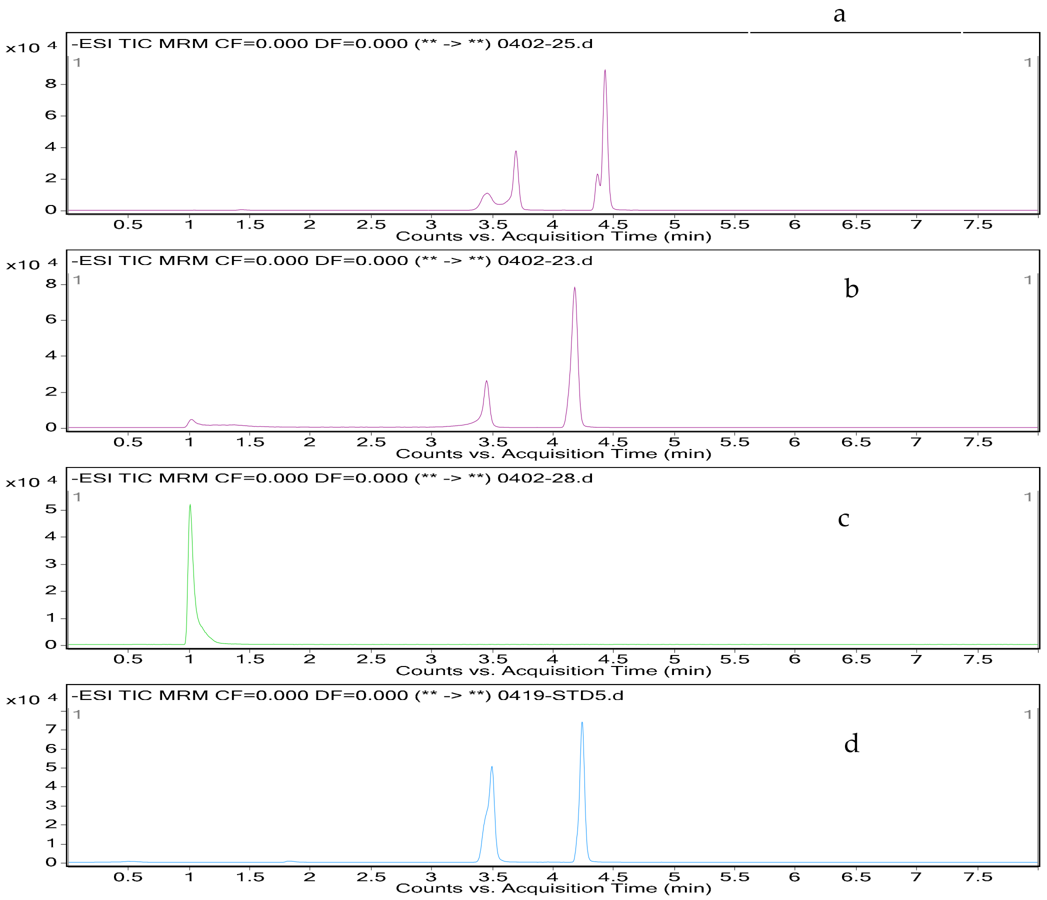

Figure S1. Chromatographic charts of three toxins separated by four chromatographic columns (column a, column b, column c, column d).

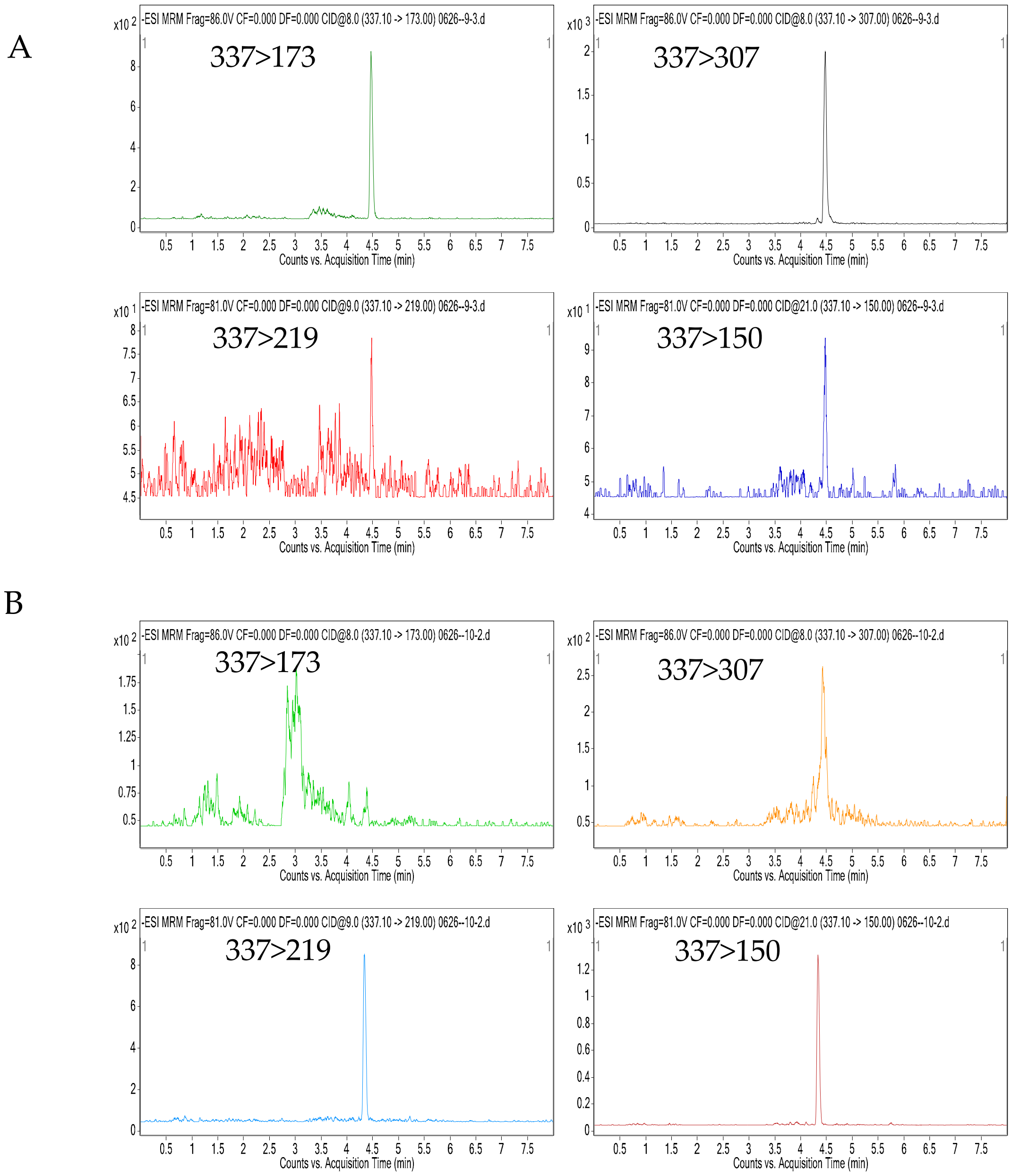

Figure S2. 3-ADON (**A**) and 15-ADON (**B**) chromatograms for negative samples.
